# Supplementary material for: End Sequence Analysis Toolkit (ESAT) expands the extractable information from single-cell RNA-seq data
Source: Genome Res. 2016 Oct;26(10):1397–410. doi: 10.1101/gr.207902.116 (PMC5052061; doi:10.1101/gr.207902.116)
Supplement: Supplemental Material [file supp_gr.207902.116_Supp_Materials.tar.gz › ESAT-esat_v0.1/doc/html/functions_func_n.html]

ESAT: Class Members - Functions


ESAT


- Main Page
- Related Pages
- Packages
- Classes
- Files

- Class List
- Class Index
- Class Hierarchy
- Class Members

- All
- Functions
- Variables
- Enumerator

- a
- b
- c
- d
- e
- f
- g
- h
- i
- j
- k
- l
- m
- n
- o
- p
- q
- r
- s
- t
- u
- v
- w
- z


All Classes Namespaces Files Functions Variables Enumerator Pages

### - n -

- NeighborAnalysis()
  : broad.pda.gene.NeighborAnalysis
- NeighborsNullModel()
  : broad.pda.gene.NeighborsNullModel
- newBase()
  : broad.core.sequence.FastaHandler
- newQuality()
  : broad.core.sequence.FastaQualityHandler
- NewRatioScore()
  : umms.core.model.score.NewRatioScore
- newSequence()
  : broad.core.sequence.FastaHandler
- newWindow()
  : broad.core.siphy.ScaledWindowParserListener
- next()
  : broad.core.datastructures.IntervalTree< V >.FwdIterator
  , broad.core.datastructures.IntervalTree< V >.OverlapIterator
  , broad.core.datastructures.IntervalTree< V >.RevIterator
  , broad.core.datastructures.JCSCache.JCSNodeIterator
  , broad.core.datastructures.ReversibleIterator< E >
  , broad.core.sequence.WindowSlider
  , broad.pda.samtools.PairedEndReadIterator
  , broad.pda.samtools.WrappedIterator
  , broad.pda.seq.fastq.FastqParser
  , broad.pda.seq.segmentation.WrappedIGVIterator
  , umms.core.coordinatesystem.ShuffledIterator< T extends Annotation >
  , umms.core.general.CloseableFilterIterator< T >
  , umms.core.model.AlignmentModel.FilteredIterator
  , umms.core.model.AlignmentModel.NodeIterator
  , umms.core.model.AlignmentModel.WrapAlignmentCountIterator
  , umms.core.model.JCSAlignmentModel.FilteredIterator
  , umms.core.model.JCSAlignmentModel.NodeIterator
  , umms.core.model.JCSAlignmentModel.WrapAlignmentCountIterator
  , umms.core.model.score.WindowScoreIterator< T extends WindowScore >
- noncentralChiSquareCdf()
  : broad.core.math.Distribution
- noncentralChiSquareDensity()
  : broad.core.math.Distribution
- norm1()
  : Jama.Matrix
- norm2()
  : Jama.Matrix
  , Jama.SingularValueDecomposition
- normalize()
  : broad.pda.seq.segmentation.ReadCountNormalizer
- normalizeBy()
  : broad.core.datastructures.MatrixWithHeaders
- normalizeByMean()
  : broad.core.datastructures.MatrixWithHeaders
- normalizeColumnsByMean()
  : broad.core.datastructures.MatrixWithHeaders
- normalizeColumnsByMedian()
  : broad.core.datastructures.MatrixWithHeaders
- normalizeReadCountsToBaseModel()
  : broad.pda.seq.segmentation.AlignmentDataModelStats
- normalizeToRelativeAbundance()
  : broad.core.math.Statistics
- normF()
  : Jama.Matrix
- normInf()
  : Jama.Matrix
- not()
  : umms.core.general.Predicates
- numberCovered()
  : broad.core.annotation.AnnotationReader< T extends GenomicAnnotation >
- numBins()
  : broad.core.math.EmpiricalDistribution
- numBlocks()
  : broad.core.sequence.SequenceRegion
  , umms.core.annotation.Annotation
  , umms.core.annotation.BasicAnnotation
  , umms.core.annotation.CompoundInterval
- numOfAnnotation()
  : broad.core.annotation.AnnotationReader< T extends GenomicAnnotation >
- numOfCompatibleIntrons()
  : umms.core.annotation.Gene
- numOfOverlapSets()
  : broad.pda.annotation.Locus
- numOverlappers()
  : broad.core.datastructures.IntervalTree< V >
- numOverlappingBases()
  : broad.core.overlaputils.GeneSetIntersect
- numOverlappingExons()
  : umms.core.annotation.Gene
- numOverlappingGenes()
  : broad.core.overlaputils.GeneSetIntersect
- numPossibleKmers()
  : broad.core.motif.SequenceMotif
- numPossibleTargets()
  : umms.core.alignment.SmatchLike


---

Generated on Fri Aug 15 2014 15:11:17 for ESAT by  

 1.8.7
